# Isolated Pediculosis Presenting With Hypereosinophilia and Iron Deficiency Anemia: A Case Report and Review of Literature

**DOI:** 10.7759/cureus.52042

**Published:** 2024-01-10

**Authors:** Susmitha Devi Chalamalasetti, Taniszahera Momin, Nicole Koutras, Juliana Cazzaniga, Premalkumar Patel

**Affiliations:** 1 Obstetrics and Gynecology, Kakatiya Medical College, Warangal, IND; 2 Medicine, Dr. Kiran C. Patel College of Osteopathic Medicine Nova Southeastern University, Clearwater, USA; 3 Medicine, Florida International University, Herbert Wertheim College of Medicine, Miami, USA; 4 Infectious Disease, Mount Sinai Medical Center, Miami, USA

**Keywords:** homelessness, microcytic anemia, iron deficiency anemia, hypereosinophilia, pediculosis

## Abstract

In recent years, there has been growing recognition of the association between lice infestation and iron deficiency anemia (IDA). The head lice, known as* Pediculosis humanus capitis,* and the body lice, known as *Pediculosis humanus corporis*, are responsible for causing these infestations. This case report focuses on the clinical findings of a 63-year-old woman who sought medical attention in the emergency department because of severe pruritus and generalized pain. Upon initial physical examination, the patient was found to have a severe lice infestation, and laboratory findings revealed IDA (Hb: 6.8 g/dL, MCV: 70 fL, iron: 21 mcg/dL) and hypereosinophilia (3,500/μL). Treatment involved the administration of Permethrin 1% lotion, Ivermectin, and intravenous iron sucrose supplementation. On the fifth day of presentation, the patient's hemoglobin level improved to 8.6 g/dL, and the eosinophil count decreased to 2,000/μL. Despite extensive investigation of the patient's IDA and hypereosinophilia, no clear etiology of IDA was identified. Consequently, the patient was diagnosed with hypereosinophilia and IDA, secondary to severe chronic pediculosis. This case report aims to raise awareness of the possibility of severe pediculosis as a risk factor for iron deficiency and hypereosinophilia.

## Introduction

Lice infestation and pediculosis are global health concerns. It is caused by a parasite belonging to the Pediculidae family, with two genera: *Pediculosis humanus* and *Phthirus pubis*. *Pediculus humanus* contains two species (*P. humanus corporis* and *P. humanus capitis*), which cause body and head lice [1,2]. The exact number of infestations per year is not known. However, it is estimated to be between six and 12 million per year among children aged three to 11 years old [3]. The third type of lice, *Phthirus pubis*, predominantly infests genital hair and is transmitted during sexual encounters [1]. Although mainly seen in the population in the first decades of their lives, it can also occur in adults who are homeless or live in shelters or refugee camps [4]. Clinical manifestations include generalized itching, excoriation, erythematous macules (feeding sites), and iron deficiency anemia (IDA) in severe cases [5,6]. This case report describes a homeless female who presented with generalized pain, pediculosis infestation, and severe pruritus and was subsequently diagnosed with IDA and eosinophilia. Limited literature exists on isolated pediculosis leading to eosinophilia and anemia. Thus, the purpose of this article is to illustrate how isolated pediculosis can manifest with marked eosinophilia and anemia.

## Case presentation

A 63-year-old homeless woman with a disheveled appearance complained of chronic fatigue and generalized pain throughout her body for one week. During the conversation, the patient was found to have tangential thoughts and a labile mood. Although the patient denied pruritus or pain caused by pruritus, she was unable to stop scratching throughout the examination. The patient denied shortness of breath, chest pain, abdominal pain, vomiting, diarrhea, hematemesis, hematochezia, hematuria, burning urination, fever, weight loss, decreased appetite, and night sweats.

Upon examination, the patient appeared pale with skin covered in hyperkeratosis and multiple excoriations. The patient's skin, hair, and clothing were heavily infested with lice and nits. Vital signs upon presentation to the emergency department included a temperature of 97.8 F, a blood pressure of 128/68 mmHg, a heart rate of 84 beats per minute, a respiratory rate of 16 breaths per minute, and a BMI of 25.6 kg/m^2^.

Initial investigations

The patient's laboratory results showed the following findings: hemoglobin level was 6.8 g/dL (reference range: 13-17 g/dL), indicating a significant decrease; mean corpuscular volume was 70 fL (reference range: 80-100 fL), indicating microcytic red blood cells; absolute eosinophil count (AEC) was elevated at 3,500/μL (reference range: 0-500/μL); serum iron level was 21 μg/dL (reference range: 50-150 μg/dL), reflecting iron deficiency; ferritin level was low at 5 ng/mL (reference range: 22-322 ng/mL), indicating reduced iron stores; transferrin saturation was only 9% (normal range: 20%-50%); and hematocrit was 22.4% (reference range: 37%-47%), indicating anemia (Table 1).

**Table 1:** Comparison of patient laboratory test results on days 1 and 5 of presentation.

Laboratory Test	Reference Range	Day 1 Result	Day 5 Result
Hemoglobin	12.0 – 16.0 g/dL	6.8 g/dL	8.6 g/dL
Mean Corpuscular Volume (MCV)	79.4 – 94.8 fL	70 fL	91.4
Absolute Eosinophil Count (AEC)	0.00 – 450/μL	3,500/μL	2,000/μL
Serum Iron	50 – 170 μg/dL	21 μg/dL	–
Ferritin	8.0 – 252.0 ng/dL	5 ng/mL	–
Transferrin Saturation	14% – 33%	9%	–
Hematocrit	37.0% – 47.0%	22.4%	30%

The patient was then hospitalized for further evaluation. Given the concerns regarding underlying psychiatric health problems, the patient was referred to psychiatry, and risperidone was initiated to address psychosis. A thorough infectious workup was conducted to investigate various parasitic and viral infections. Blood and stool cultures were performed and remained sterile on day 5. Stool testing for ova and parasites for three consecutive days remained negative. Serology for strongyloides, HIV, and hepatitis was all negative. Peripheral blood smear reviewed by the pathologist showed no abnormal cells.

At the suggestion of the hematology-oncology team, a pan CT scan of the body was performed to assess organ involvement due to eosinophilia; however, no significant pathology was detected. Laboratory findings included various parameters related to the patient's blood, such as hemoglobin level, mean corpuscular volume (MCV), AEC, serum iron level, ferritin level, transferrin saturation, and hematocrit. Reference ranges for each parameter were also provided for the context.

Treatment

After showering, the patient's clothing and bedding were replaced. A 1% lotion of permethrin was applied to the hair and scalp, and a repeat permethrin application was advised one week later. Ivermectin 200 mcg/kg was administered to treat pediculosis, after which the patient's eosinophil count began to decrease. The patient was prescribed an iron sucrose injection of 200 mg intravenous daily for five days to address IDA.

Outcome and follow-up

The hemoglobin level improved to 8.6 g/dL and her eosinophil count decreased to 2,000/microliter on day 5 of presentation. The patient was discharged from the hospital on oral iron for anemia and was advised to follow-up with primary care and psychiatric outpatient services.

## Discussion

Along with the population in the first decade of their lives, pediculosis is also prevalent in the homeless population owing to a lack of hygiene. Transmission occurs via physical contact with individual or personal items such as sharing a bed. Generalized pruritus is the most common clinical presentation and is thought to be due to an allergic reaction to the louse’s salivary antigens [7].

This allergic reaction is also hypothesized to be the cause of the increase in the number of eosinophils in lice-infested individuals [8]. There is limited research and literature available regarding hypereosinophilia and pediculosis in humans; therefore, we presume that she has a similar reason for hypereosinophilia based on her severe pediculosis and negative work-up for other possible causes. An experimental study in pigs showed higher circulating eosinophil counts in pigs that were infested with Haematopinus suis (the hog louse) than in uninfested controls [9].

**Figure 1:**
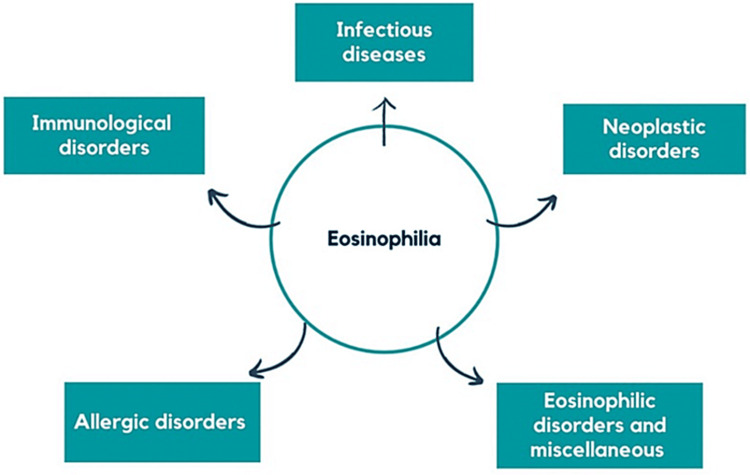
Overview of eosinophilia causes. Image Credits: Susmita Devi Chalamalasetti

Eosinophilia is defined as ≥500 eosinophils/µL, and hypereosinophilia is defined as ≥1,500 eosinophils/µL [10]. Eosinophilia may be caused by numerous conditions, including allergic diseases, medication reactions, infectious diseases (such as helminth, protozoan, viral, and fungal infections), inflammatory disorders, and neoplastic disorders (Figure 1) [10].

Eosinophils, an important type of white blood cell involved in disease defense, can become elevated (eosinophilia) when exceeding 500 cells/µL of blood owing to various conditions [10]. This figure presents the primary categories of factors contributing to eosinophilia, including immunological disorders, allergic disorders, infectious diseases, neoplastic disorders, eosinophilic disorders, and miscellaneous factors [10]. A diagram was created by the author using Canvas, a platform provided by Google.

Although the exact relationship between lice infestation, IDA, and eosinophilia has not been clearly established, multiple case reports suggest that heavy infestation can result in IDA caused by chronic, low-volume blood loss, which is sometimes associated with eosinophilia, although rarely with such high eosinophil counts, as observed in our patient. One study reported an approximate blood loss of 0.7 mL/day in an infected individual who had 2,657 lice [11]. Another retrospective study also found severe IDA (<6 g/dL) in all homeless patients with reported lice infestation, despite no blood loss and negative endoscopy/colonoscopy findings [6]. In our patient, hematology-oncology was consulted and due to the lack of abnormal cells on peripheral blood smear, a bone marrow biopsy was not indicated. Thus, it can be inferred that the combination of blood loss, inadequate nutrition, psychopathology, and behavioral disturbances, as observed in our patient, can lead to severe IDA.

Table 2 presents a few cases of Pediculosis manifestations associated with profound IDA, with hemoglobin values ranging from 3.2 g/dL to 6.3 g/dL and eosinophilia ranging from 0 to 3,000/μL when the workup was otherwise normal. The fact that there was only one case of eosinophilia in the table above with an eosinophil count above 1,500/μL indicates that the combination of hypereosinophilia and anemia with isolated pediculosis is rare and requires further study.

**Table 2:** Hematological parameters in various cases of pediculosis. Reference range: Hemoglobin Male: 13.5-17.5 g/dL and Female: 12.0-15.5 g/dL; Ferritin Male: 24-336 ng/dL and Female: 11-307 ng/dL

Study	Age (years)	Gender	Hemoglobin	Ferritin	Absolute Eosinophil Count
Woodruff et al. 2019 [2]	13	Female	3.8 g/dL	5 ng/dL	0.54 k/μL
Gus et al. 2011 [5]	55	Female	3.2 g/dL	5 ng/dL	0 k/μL
	61	Male	4.0 g/dL	6 ng/dL	3.0 k/μL
	43	Male	5.7 g/dL	19 ng/dL	1.4 k/μL
	52	Male	4.7 g/dL	6 ng/dL	0 k/μL
	50	Male	4.4 g/dL	8 ng/dL	0.77 k/μL
	54	Female	5.7 g/dL	11 ng/dL	0.03 k/μL
Batool et al. 2021 [12]	32	Male	6.3 g/dL	25 ng/dL	0.82 k/μL
						Chalamalasetti et al. 2023 (Current article)	63	Female	6.8 g/dL	5 ng/dL	3.5k/μL

The table provides hematological parameters in cases with pediculosis manifestations across three different studies. Reference ranges for the parameters are as follows: Hemoglobin levels are typically between 13.5 and 17.5 g/dL for males and 12.0-15.5 g/dL for females; ferritin levels generally range from 24 to 336 ng/dL for males and 11-307 ng/dL for females; the normal range for AEC is 0.04-0.4 k/μL.

These references provide context to the measurements in the table and offer a baseline for understanding the significance of the data presented. Please note that the reference ranges for hemoglobin and ferritin may slightly vary depending on the laboratory, but these are commonly accepted ranges.

In our patient, a thorough infectious workup was performed, including blood culture, stool studies, and serological tests for various parasitic and viral infections, all of which yielded negative results. Eosinophilia >1,500/μL increases the risk of organ involvement [13]. A pan-CT of the body was performed to rule out organ involvement, but no significant pathology was found [14]. Considering that the patient had no symptoms, imaging, or laboratory findings indicating any other immunological, allergic, infectious, or neoplastic causes of hypereosinophilia and anemia, and given the presence of numerous lice and nits have been found on the skin, hair, and clothing, it is reasonable to deduce pediculosis as a possible cause of the significant anemia and eosinophilia.

## Conclusions

In conclusion, severe pediculosis infestation can lead to a range of complications. Despite minimal blood loss, severe chronic infestation can be associated with IDA, as presented in our case. Poor nutritional status and chronic low-volume blood loss are possible mechanisms for IDA in this population. Isolated pediculosis rarely leads to a combination of hypereosinophilia and anemia. However, this possibility should be considered in patients with pediculosis.
